# Tick saliva-induced programmed death-1 and PD-ligand 1 and its related host immunosuppression

**DOI:** 10.1038/s41598-020-80251-y

**Published:** 2021-01-13

**Authors:** Yamato Sajiki, Satoru Konnai, Yoshinori Ikenaka, Kevin Christian Montecillo Gulay, Atsushi Kobayashi, Luís Fernando Parizi, Benvindo Capela João, Kei Watari, Sotaro Fujisawa, Tomohiro Okagawa, Naoya Maekawa, Carlos Logullo, Itabajara da Silva Vaz, Shiro Murata, Kazuhiko Ohashi

**Affiliations:** 1 Department of Disease Control, Faculty of Veterinary Medicine, Hokkaido University, Kita 18, Nishi 9, Kita-ku, Sapporo, 060-0818 Japan; 2 Department of Advanced Pharmaceutics, Faculty of Veterinary Medicine, Hokkaido University, Sapporo, 060-0818 Japan; 3 Department of Environmental Veterinary Sciences, Faculty of Veterinary Medicine, Hokkaido University, Sapporo, 060-0818 Japan; 4 Department of Veterinary Clinical Medicine, Faculty of Veterinary Medicine, Hokkaido University, Sapporo, 060-0818 Japan; 5 Centro de Biotecnologia, Universidade Federal do Rio Grande do Sul, Porto Alegre, RS 91501-970 Brazil; 6 Laboratório Integrado de Bioquímica Hatisaburo Masuda and Laboratório Integrado de Morfologia, NUPEM-UFRJ, Macaé, RJ Brazil

**Keywords:** Immunology, Pathogenesis

## Abstract

The tick *Rhipicephalus microplus* is a harmful parasite of cattle that causes considerable economic losses to the cattle breeding industry. Although *R*. *microplus* saliva (Rm-saliva) contains several immunosuppressants, any association between Rm-saliva and the expression of immunoinhibitory molecules, such as programmed death (PD)-1 and PD-ligand 1 (PD-L1), has not been described. In this study, flow cytometric analyses revealed that Rm-saliva upregulated PD-1 expression in T cells and PD-L1 expression in CD14^+^ and CD11c^+^ cells in cattle. Additionally, Rm-saliva decreased CD69 expression in T cells and Th1 cytokine production from peripheral blood mononuclear cells. Furthermore, PD-L1 blockade increased IFN-γ production in the presence of Rm-saliva, suggesting that Rm-saliva suppresses Th1 responses via the PD-1/PD-L1 pathway. To reveal the upregulation mechanism of PD-1/PD-L1 by Rm-saliva, we analyzed the function of prostaglandin E_2_ (PGE_2_), which is known as an inducer of PD-L1 expression, in Rm-saliva. We found that Rm-saliva contained a high concentration of PGE_2_, and PGE_2_ treatment induced PD-L1 expression in CD14^+^ cells in vitro. Immunohistochemical analyses revealed that PGE_2_ and PD-L1 expression was upregulated in tick-attached skin in cattle. These data suggest that PGE_2_ in Rm-saliva has the potential to induce the expression of immunoinhibitory molecules in host immune cells.

## Introduction

The *Rhipicephalus microplus* is a one-host tick that feeds on cattle. It is one of the most harmful parasites of cattle in subtropical areas of the world and causes significant economic losses to the cattle industry^1^. The tick causes many deleterious effects including loss of blood, slowed weight gain, and reduced milk production^1^. Additionally, the tick transmits *Babesia* spp. and *Anaplasma* spp. to cattle^2,3^. Although chemical acaricides are used to control tick infestation, the continuous use of these acaricides causes many adverse side effects including the selection of resistant tick populations to these chemicals as well as food and environmental contamination^4,5^. Thus, the development of an alternative control strategy, such as a tick vaccine^6^, is strongly needed.

Tick saliva is important to obtain a blood meal and facilitate pathogen transmission to the vertebrate host. Tick saliva contains bioactive factors including a large variety of immunosuppressants^7^, such as cystatins^8^, serpins^9^, metalloproteinases^10^, and lipocalins^11^. For example, salivary cystatins, such as sialostatin L and sialostatin L2, suppress host immune responses, leading to the enhancement of tick-borne pathogen transmission^8,12,13^. Interestingly, Kotsyfakis et al. have reported that guinea pig vaccination against sialostatin L2 leads to a decreased feeding ability and increased rejection rate of *Ixodes scapularis* nymphs^14^. Hence, salivary immunosuppressants are a potential target for tick control. Previous studies on *R*. *microplus* have described that host exposure to salivary gland extracts regulates immune responses such as macrophage activation^15^. Furthermore, isolated salivary proteins modulate the immune response^9,13,16–18^. However, the detailed mechanism of immunomodulation in cattle caused by *R*. *microplus* saliva (Rm-saliva) is not well understood.

Prostaglandin E_2_ (PGE_2_) is an inflammatory mediator derived from arachidonic acid by several enzymes such as cyclooxygenase (COX)-1 and COX-2^19^. PGE_2_ regulates the activity of immune cells, such as T cells, dendritic cells (DCs), and macrophages, contributing to immune evasion during cancer and chronic infection^20^. PGE_2_ inhibits Th1 immune responses, including T-cell proliferation and Th1 cytokine production in cattle^21^. Interestingly, several tick genera, such as *Amblyomma* spp., *Ixodes* spp., and *Rhipicephalus* spp., secrete PGE_2_ in their saliva^22–25^. The immunomodulatory effects of PGE_2_ in tick saliva have been reported recently^26–28^. For instance, Sá-Nunes et al. have shown that PGE_2_ in *I*. *scapularis* saliva inhibits DC function and maturation in vitro^26^. Although Rm-saliva contains PGE_2_^29,30^, its effects on bovine immune cells were still unclear. Furthermore, PGE_2_ induces programmed death (PD)-1 and PD-ligand 1 (PD-L1) expression in several animal models including cattle^21,31–33^. PD-1 is an immune checkpoint molecule that is expressed on T cells, and negatively regulates T-cell activation via interaction with its ligands PD-L1 and PD-L2. Therefore, PD-1 upregulation plays a key role in T-cell exhaustion^34^. Numerous studies have revealed that tick saliva inhibits T-cell activation, especially Th1 cytokine production^35,36^. However, little information is available on the association of tick saliva with the expression of immunoinhibitory molecules.

Here, we analyzed the expression of PD-1 and PD-L1 in bovine immune cells in the presence of Rm-saliva. We then examined PGE_2_ concentrations in Rm-saliva by enzyme-linked immunosorbent assay (ELISA) and ultra-performance liquid chromatography (UPLC)–mass spectrometry (MS)/MS system, and we examined the association of PGE_2_ in Rm-saliva with PD-L1 expression by in vitro culture and immunohistochemistry.

## Results

### Upregulation of PD-1/PD-L1 expression by Rm-saliva

**Figure 1:**
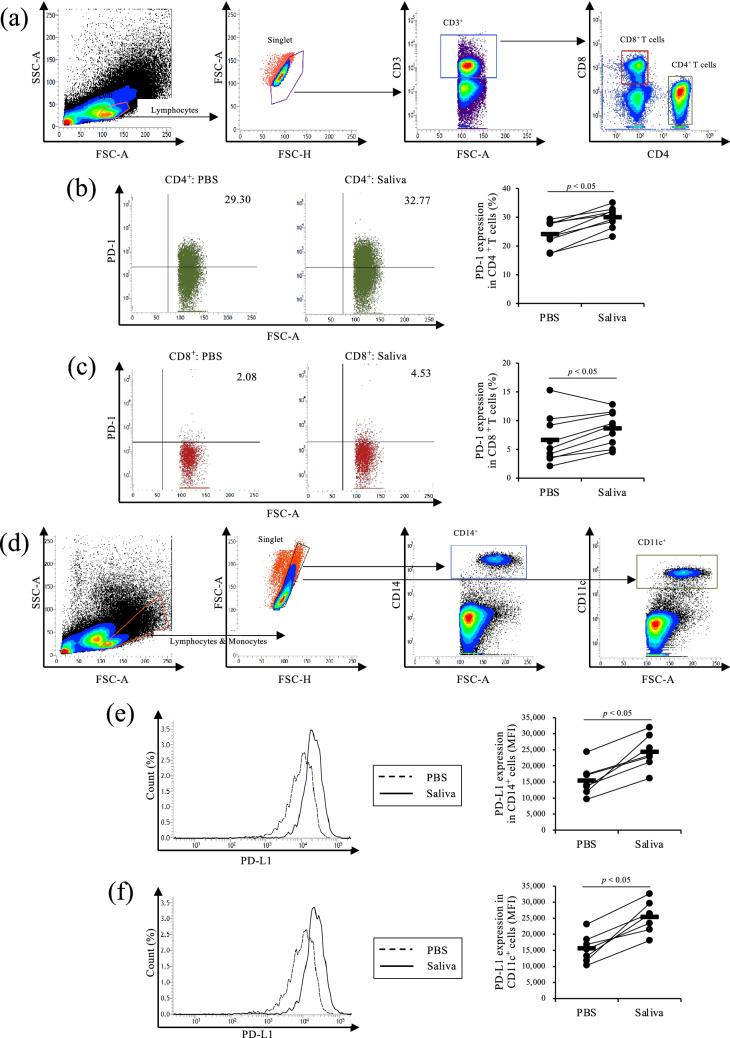
Upregulation of PD-1 and PD-L1 expression by *Rhipicephalus microplus* saliva (Rm-saliva). (**a**–**f**) PBMCs were cultured with Rm-saliva. (**a**) Gating strategy for PD-1 staining. (**b**,**c**) PD-1 expression in CD4^+^ (**b**) and CD8^+^ (**c**) T cells was measured by flow cytometry. (**d**) Gating strategy for PD-L1 staining. (**e**,**f**) PD-L1 expression in CD14^+^ (**e**) and CD11c^+^ (**f**) cells was measured by flow cytometry. (**a**–**f**) Statistical difference was identified by the Wilcoxon signed-rank test. *MFI* mean fluorescence intensity.

To examine whether Rm-saliva induces PD-1 and PD-L1 expression in cattle, peripheral blood mononuclear cells (PBMCs) were cultured with Rm-saliva. PD-1 expression in CD4^+^ and CD8^+^ T cells was increased by Rm-saliva (Fig. 1a–c). Additionally, PD-L1 expression levels in CD14^+^ and CD11c^+^ cells were also increased by Rm-saliva (Fig. 1d–f). These results suggest that Rm-saliva is an inducer of immunoinhibitory molecules, such as PD-1 and PD-L1, in bovine immune cells.

### Inhibition of Th1 responses by Rm-saliva via PD-1/PD-L1 pathway

**Figure 2:**
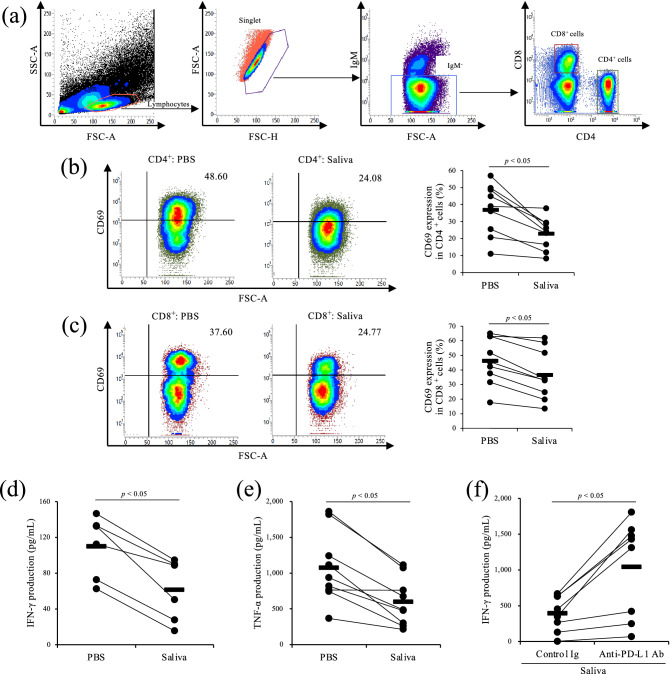
Inhibition of Th1 responses by Rm-saliva. (**a**–**e**) PBMCs were cultured with Rm-saliva in the presence of anti-CD3 and anti-CD28 mAbs. (**a**) Gating strategy for CD69 expression. (**b**,**c**) CD69 expression in CD4^+^ (**b**) and CD8^+^ (**c**) cells was measured by flow cytometry. (**d**,**e**) IFN-γ (**d**) and TNF-α (**e**) concentrations in culture supernatants were determined by ELISA. (**f**) PBMCs were cultured with anti-PD-L1 Ab (Boch4G12) in the presence of Rm-saliva. Bovine IgG was used as a control Ab. PBMCs were stimulated by anti-CD3 and anti-CD28 mAbs. IFN-γ concentration was determined by ELISA. (**a**–**f**) Statistical difference was identified by the Wilcoxon signed-rank test.

In cattle, the PD-1/PD-L1 pathway is involved in the inhibition of Th1 immune responses, such as IFN-γ production^37^. Therefore, to analyze whether Rm-saliva inhibits bovine Th1 responses, we cultured bovine PBMCs with Rm-saliva in the presence of T-cell stimulation. Treatment with Rm-saliva downregulated the expression of CD69, an activation marker, in CD4^+^ and CD8^+^ cells (Fig. 2a–c). Furthermore, treatment with Rm-saliva decreased IFN-γ and TNF-α production from bovine PBMCs (Fig. 2d,e). Interestingly, the blockade of the PD-1/PD-L1 pathway using anti-PD-L1 antibodies (Abs) enhanced IFN-γ production in the presence of Rm-saliva (Fig. 2f), suggesting that Rm-saliva inhibits bovine Th1 responses, at least in part, via the PD-1/PD-L1 pathway.

### Induction of PD-L1 expression via PGE_2_ in Rm-saliva

**Figure 3:**
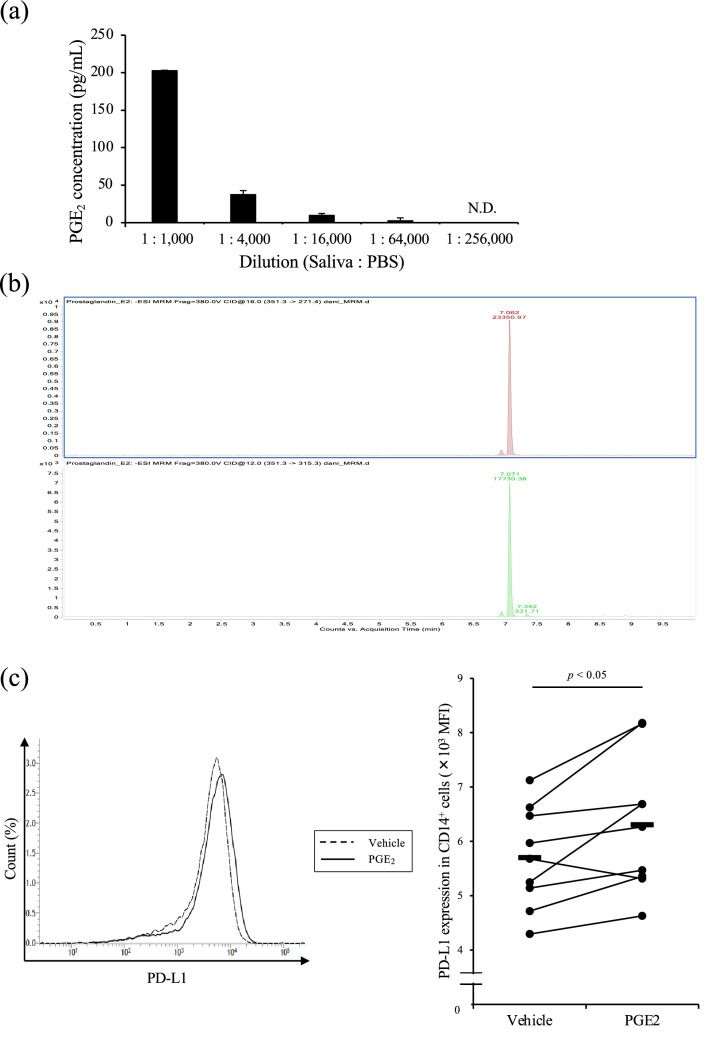
The presence of PGE_2_ in Rm-saliva. (**a**) PGE_2_ concentration of Rm-saliva was measured by ELISA. (**b**) The purification of PGE_2_ from Rm-saliva was conducted by SPE combined with fractionation using HPLC. (**c**) CD14^+^ cells were cultured with PGE_2_, and PD-L1 expression was measured by flow cytometry. Statistical difference was identified by the Wilcoxon signed-rank test. *N.D.* not detected.

**Figure 4:**
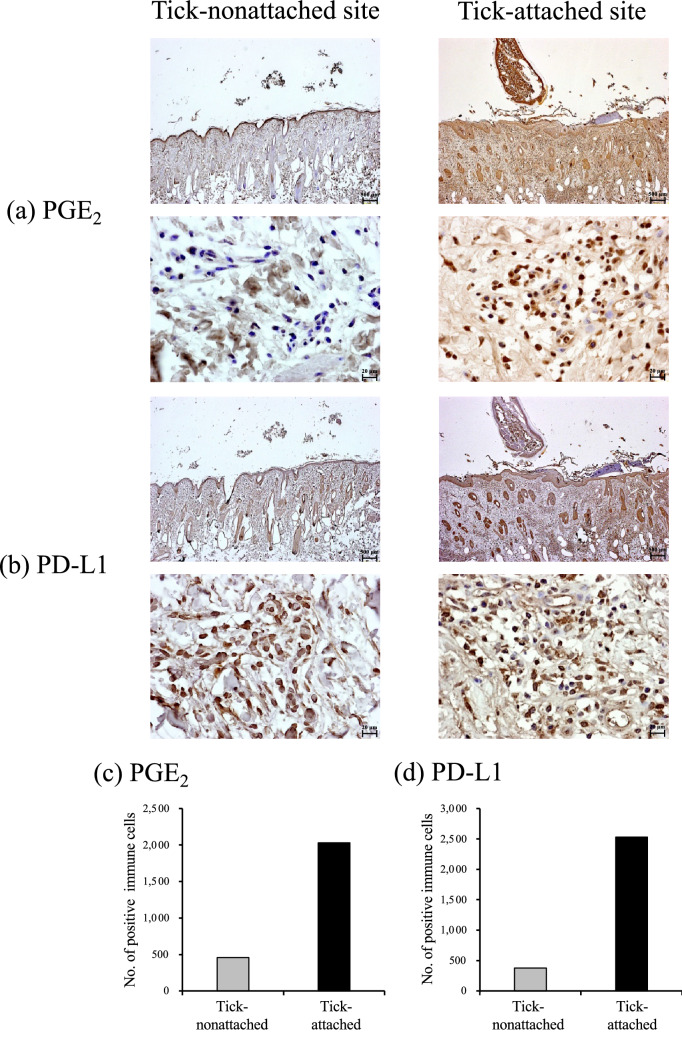
PGE_2_ and PD-L1 expression in *R*. *microplus*-attached and nonattached sites. (**a**,**b**) Immunohistochemical staining of PGE_2_ (**a**) and PD-L1 (**b**) in tick-attached and nonattached sites on cattle were performed using anti-human PGE_2_ antibody (rabbit polyclonal) and anti-bovine PD-L1 mAb (6C11-3A11). (**c**,**d**) The number of PGE_2_ (**c**) and PD-L1 (**d**) positive immune cells in the sample areas.

We then demonstrated the upregulation mechanism of PD-1/PD-L1 expression by Rm-saliva. The concentration of PGE_2_ in Rm-saliva determined by ELISA was approximately 200 ng/mL (Fig. 3a). In addition, Rm-saliva was also qualified and quantified by UPLC-MS/MS system. A clear single peak was confirmed in the transition of *m/z* 351 > 271 (qualifier) and *m/z* 351 > 315 (quantifier) at the same retention time to the PGE_2_ standard (7.1 min) (Fig. 3b). The estimated concentration of PGE_2_ from Rm-saliva using an external calibration curve was 330 ng/mL. We previously have reported that PGE_2_ induces PD-L1 expression in bovine immune cells^21,33^. In the present study, bovine CD14^+^ cells were cultured with 100 ng/mL of PGE_2_. The concentration of PGE_2_ in the cell culture was similar to that in Rm-saliva. As shown in Fig. 3c, PD-L1 expression in CD14^+^ cells was significantly upregulated by PGE_2_ treatment in vitro (Fig. 3c). In addition, immunohistochemical staining was performed to examine PGE_2_ and PD-L1 expression in tick-attached and tick-nonattached sites. We observed the increase in both PGE_2_ positive and PD-L1 positive immune cells in tick-attached site, although statistical analysis was not done because of the limited number of samples that were evaluated. (Fig. 4a–d). Thus, PGE_2_ in Rm-saliva may be associated with the upregulation of PD-1/PD-L1 expression.

## Discussion

The immunomodulatory properties of tick saliva have been investigated in several in vitro and in vivo studies. The saliva compounds such as serpins^9,38^, cystatins^39,40^, PGE_2_^26,27^, and other molecules^41,42^ can interfere with different host immune cells to modulate the host immune response and to help tick feeding.

In the present study, we demonstrated the existence of PGE_2_ in Rm-saliva. PGE_2_ concentration in Rm-saliva (200 ng/mL) was much higher (approximately 2740-fold greater) than that in the plasma from healthy cattle (73 pg/mL) as described in a previous paper^43^. We previously showed that PGE_2_ treatment in vitro suppressed Th1 cytokine production and T-cell proliferation in cattle^21^. Thus, salivary PGE_2_ is identified as one of the immunosuppressants in *R*. *microplus*. PGE_2_ has immunosuppressive effects not only on T cells, but also on other immune cells, such as macrophages and DCs^20^. PGE_2_ in *I*. *scapularis* saliva inhibits DC maturation and function^26^, and PGE_2_ in *Dermacentor variabilis* saliva regulates macrophage activity^27^. Additionally, Rm-saliva is involved in immunoregulation of bovine macrophages^15^. Furthermore, the data obtained from the skin biopsies of tick-resistant and tick-susceptible cattle have shown that innate and adaptive immune pathways are activated in tick-resistant animals and therefore could be modulated by tick saliva to the benefit of the feeding ticks^44^. Together, these findings suggest the contribution of the PGE_2_ in tick saliva to the parasitism by modulating both the innate immune responses, such as the activities of DCs and macrophages^25–28^, and adaptive immune responses (present study) in vitro and in vivo. However, additional experiments are necessary to examine the association of PGE_2_ in Rm-saliva with other bovine immune cells.

The PD-1/PD-L1 pathway contributes to the process of T-cell exhaustion^34^. A previous study has shown that a salivary gland protein from *R*. *appendiculatus* upregulates PD-L1 expression in human DCs^45^. Carvalho-Costa et al. also have shown that *Amblyomma cajennense* tick saliva increases PD-L1 expression in murine bone marrow-derived DCs^46^. However, to date, no report demonstrates the association of tick saliva with immunosuppression caused by the PD-1/PD-L1 pathway. In the present study, we found that Rm-saliva induced PD-1 expression in T cells and PD-L1 expression in CD14^+^ and CD11c^+^ cells in vitro. The blockade of PD-1/PD-L1 interaction using an anti-PD-L1 Ab ameliorated the Rm-saliva-induced Th1 suppression. To the best of our knowledge, this is the first study that shows that tick saliva regulates host immune responses, at least in part, via PD-1/PD-L1 upregulation. Our previous studies have demonstrated that PGE_2_ is one of the inducers of PD-L1 expression in bovine CD14^+^ cells^21,33^. In the present study, we found that Rm-saliva contained a high concentration of PGE_2_. In addition, the treatment with commercially available PGE_2_ increased PD-L1 expression in CD14^+^ cells, suggesting that Rm-saliva upregulates PD-L1 expression via PGE_2_. To reveal the direct relationship between PD-L1 upregulation and PGE_2_ derived from Rm-saliva, we attempted to purify PGE_2_ from Rm-saliva. However, PD-L1 upregulation by PGE_2_ derived from Rm-saliva was not confirmed in the present study, presumably due to contamination by an acidic solvent during the purification process (data not shown). In addition, we also attempted to elucidate the direct relationship by using the blockers of PGE_2_ receptors. However, it was not able to demonstrate using the in vitro cultures because treatment with PGE_2_ receptor blockers in vitro increased PD-L1 expression presumably due to the induction of cytokine production such as IFN-γ and TNF-α (data not shown). Therefore, to establish an appropriate evaluation system is required, and future studies will examine in details the biological effect of PD-L1 upregulation induced by PGE_2_ from Rm-saliva in the host–parasite relationship. Further, recent studies in human research have shown that PGE_2_ also induces the expression of other immunoinhibitory molecules, such as PD-1 and T-cell immunoglobulin and mucin domain 3^32,47^. Our data demonstrated that Rm-saliva induced PD-1 expression in bovine T cells in vitro. However, it is still unclear whether PGE_2_ in tick saliva is involved in the upregulation of other immunoinhibitory molecules. Additional experiments to address the issue should be conducted in the future research.

Previous studies have shown the correlations between the expression of immunoinhibitory receptors in bovine T cells and Th1 responses, such as IFN-γ production. A study showed that PD-1 expression in CD4^+^ and CD8^+^ T cells was upregulated in cattle infected with *Mycoplasma bovis*, and negative correlations were observed between PD-1 expression in T cells and IFN-γ production from PBMCs^48^. Similarly, another study showed that the expression of lymphocyte activation gene-3 (LAG-3), an immunoinhibitory molecule expressed on T cells, was upregulated in T cells of bovine leukemia virus-infected cattle, and the expression levels of LAG-3 in CD4^+^ T cells were negatively correlated with the expression of *IFN-γ* gene^49^. Here, by treatment with Rm-saliva, the percentage of PD-1^+^CD4^+^ and PD-1^+^CD8^+^ T cells was increased significantly but not dramatically. However, based on these previous studies, the differences observed in Fig. 1b,c could have influence in the activity of T cells.

Tick saliva potentiates infection with a variety of agents that cause tick-borne diseases^50^, but detailed mechanisms of heightened infectivity have not been fully elucidated. The modulation of host immune systems is speculated as a critical element^51,52^. Th1 responses help to protect the host against tick-borne pathogens, including *Anaplasma marginale*, which is transmitted to cattle by *R*. *microplus*^53,54^. In addition, the PD-1/PD-L1 pathway is used as an immune evasion mechanism of *A*. *marginale* from host defense^55^. Interestingly, PD-L1 has been used in a chimeric vaccine against *Babesia microti* infections in which the extracellular domain of PD-L1 was fused to the C-terminus of *B. microti*-profilin^56^. Our results demonstrated that Rm-saliva regulates immune responses, especially Th1 responses, through PD-1/PD-L1 upregulation, suggesting that Rm-saliva promotes the transmission of tick-borne pathogens via PD-1/PD-L1-mediated Th1 suppression.

In conclusion, the results of the present study revealed the association of Rm-saliva with PD-1/PD-L1 upregulation in host immune cells. Rm-saliva regulated Th1 immune responses by the PD-1/PD-L1 pathway. Additionally, Rm-saliva contained a high concentration of PGE_2_, and treatment with PGE_2_ in vitro induced PD-L1 expression in bovine CD14^+^ cells. Furthermore, immunohistochemical analyses showed that the expression of both PGE_2_ and PD-L1 was upregulated in the tick-attached site, suggesting that PGE_2_ in Rm-saliva may increase PD-L1 expression. Further studies will examine the role of Rm-saliva-induced PD-1/PD-L1 upregulation on the transmission of tick-borne pathogens.

## Methods

### Ticks and saliva collection

*Rhipicephalus* *microplus* ticks (Porto Alegre strain, free from *Babesia* spp. and *Anaplasma* spp.) were obtained from a laboratory colony maintained as previously described^57^. Hereford calves were acquired from a tick-free area and infested with 10-day-old *R*. *microplus* larvae. Fully engorged female ticks were obtained after the spontaneous detachment from the calves. Ticks were rinsed with sterile distilled water, and salivation was induced by dorsal injection of 5 µL of 2% pilocarpine hydrochloride (Sigma-Aldrich, St. Louis, MO, USA) in ethanol (Merck KGaA, Darmstadt, Germany)^58,59^. Rm-saliva was collected using a pipette tip from ticks maintained at 28 °C in a humid chamber for approximately 3 h. Collected Rm-saliva was stored at − 80 °C upon use in the experiments. Animals used in these experiments were housed in Faculdade de Veterinária, Universidade Federal do Rio Grande do Sul (UFRGS), Brazil. Experiments were conducted considering ethical and methodological guidelines in agreement with the Norms by the Animal Experimentation Ethics Committee of the UFRGS. The protocols were approved by the Comissão de Ética no Uso de Animais (CEUA)-UFRGS. These experiments were carried out in compliance with the ARRIVE guidelines (http://www.nc3rs.org.uk/page.asp?id=1357).

### Blood collection and cell preparation

Bovine blood samples were collected by veterinarians and PBMCs were separated from the blood samples using density-gradient centrifugation on Percoll (GE Healthcare, Little Chalfont, UK). The experiments using bovine blood samples were approved by the Ethics Committee of the Faculty of Veterinary Medicine, Hokkaido University (approval numbers: 17-0014 and 17-0024). These experiments were carried out in compliance with the ARRIVE guidelines (http://www.nc3rs.org.uk/page.asp?id=1357). To isolate CD14^+^ cells from PBMCs, PBMCs were cultured with anti-bovine CD14 monoclonal antibody (mAb) (CAM36A; WSU Monoclonal Antibody Center, Pull-man, WA, USA) for 30 min at 4 °C. Cells were then incubated with anti-mouse IgG_1_ MicroBeads (Miltenyi Biotec, Bergisch Gladbach, Germany) for 15 min at 4 °C. After incubation, CD14^+^ cell sorting was conducted using an autoMACS Pro (Miltenyi Biotec) according to the manufacturer’s protocol. The purity of the CD14^+^ cells (> 90%) was confirmed using FACS Verse (BD Biosciences, San Jose, CA, USA). Cells were cultured in RPMI 1640 medium (Sigma-Aldrich) containing 10% heat-inactivated fetal calf serum (Thermo Fisher Scientific, Waltham, MA, USA), 100 U/mL penicillin (Thermo Fisher Scientific), 100 μg/mL streptomycin (Thermo Fisher Scientific), and 2 mM l-glutamine (Thermo Fisher Scientific) using 96-well plates (Corning Inc., Corning, NY, USA).

### PBMC culture

PBMCs were cultured with 1 µL of Rm-saliva in 100 µL of culture medium for 24 or 72 h. Cultures were stimulated with or without 1 μg/mL of anti-CD3 mAb (MM1A; WSU Monoclonal Antibody Center) and 1 μg/mL of anti-CD28 mAb (CC220; Bio-Rad, Hercules, CA, USA). After 24 h of incubation with the stimulation, PBMCs were collected, and CD69 expression was examined by flow cytometry as described below. After 72 h of incubation with the stimulation, culture supernatants were collected and the concentrations of IFN-γ and TNF-α were measured by ELISA as described below. After 24 h of incubation without the stimulation, the cells were collected, and the expression of PD-1 and PD-L1 was measured by flow cytometry as described below.

To examine whether the inhibition of the PD-1/PD-L1 pathway using a specific antibody (Ab) rescues IFN-γ production in the presence of Rm-saliva, PBMCs were cultured with 1 µL of Rm-saliva and 10 µg/mL of anti-PD-L1 Ab (Boch4G12)^60^ in the presence of 1 μg/mL of anti-CD3 mAb and 1 μg/mL of anti-CD28 mAb in 100 µL of culture medium for 72 h. Bovine IgG (Sigma-Aldrich) was used as a negative control. After incubation, culture supernatants were collected, and IFN-γ concentrations were measured by ELISA as described below.

### CD14^+^ cell culture

Isolated CD14^+^ cells were cultured with 100 ng/mL of PGE_2_ (Cayman Chemical, Ann Arbor, MI, USA) for 24 h. Dimethyl sulfoxide (DMSO; Nacalai Tesque, Kyoto, Japan) was used as a vehicle control. After incubation, cells were collected and PD-L1 expression was measured by flow cytometry as described in the following section.

### Flow cytometry

For Fc blocking, PBMCs were incubated with phosphate buffered saline (PBS) including 10% goat serum (Thermo Fisher Scientific) for 15 min at 25 °C to prevent nonspecific reactions. For PD-1 staining, cells were stained after Fc blocking by anti-bovine PD-1 mAb (5D2, rat IgG_2a_)^61^ or rat IgG_2a_ isotype control (R35-95; BD Biosciences) for 30 min at 37 °C. After washing twice with PBS containing 1% bovine serum albumin (BSA, Sigma-Aldrich), the cells were stained with PerCP/Cy5.5-conjugated anti-CD3 mAb (MM1A), FITC-conjugated anti-CD4 mAb (CC8; Bio-Rad), PE-conjugated anti-CD8 mAb (CC63; Bio-Rad), and APC-conjugated anti-rat immunoglobulin antibody (Southern Biotech, Birmingham, AL, USA) for 20 min at 25 °C. MM1A was conjugated using a Lightning-Link antibody labeling kit (Innova Biosciences, Cambridge, England, UK). For PD-L1 staining, PBMCs and CD14^+^ cells were stained after Fc blocking using anti-bovine PD-L1 mAb (4G12, rat IgG_2a_)^62^ for 30 min at 37 °C. Rat IgG_2a_ isotype control was used as an isotype control. After washing twice with PBS containing 1% BSA, the cells were stained by APC-conjugated anti-rat immunoglobulin antibody for 20 min at 25 °C. For PBMC staining, the cells were also stained with PerCP/Cy5.5-conjugated anti-CD14 mAb (CAM36A) and PE-labeled anti-CD11c mAb (BAQ151A; WSU Monoclonal Antibody Center) at this step. CAM36A was prelabeled using the Lightning-Link antibody labeling kit. BAQ151A was prelabeled with a Zenon Mouse IgG_1_ Labeling Kit (Thermo Fisher Scientific). After the final staining, the cells were washed twice with PBS containing 1% BSA, and analyzed immediately by FACS Verse.

To measure CD69 expression, PBMCs were stained after Fc blocking with FITC-conjugated anti-CD4 mAb (CC8), PE-conjugated anti-CD8 mAb (CC63), PE/Cy7-conjugated anti-IgM mAb (IL-A30; Bio-Rad), and Alexa Fluor 647-labeled anti-CD69 mAb (KTSN7A; Kingfisher Biotech, St. Paul, MN, USA). IL-A30 was prelabeled using the Lightning-Link antibody labeling kit. KTSN7A was prelabeled with the Zenon Mouse IgG_1_ Labeling Kit. After the staining, stained cells were washed twice with PBS containing 1% BSA, and analyzed immediately by FACS Verse.

### ELISA

IFN-γ and TNF-α concentrations in culture supernatants collected from PBMC cultures were determined by Bovine IFN-γ ELISA Development Kit (Mabtech, Nacka Strand, Sweden) and Bovine TNF-α Do-It-Yourself ELISA (Kingfisher Biotech), respectively, according to the manufacturers’ instructions. The PGE_2_ concentration in Rm-saliva was measured by Prostaglandin E_2_ Express ELISA Kit (Cayman Chemical) per the manufacturer’s instruction.

### PGE_2_ purification

PGE_2_ purification from Rm-saliva was conducted by the combination with solid phase extraction (SPE) and the fractionation by using high-performance liquid chromatography (HPLC). The SPE method was performed as previously described^63^. Briefly, 2 mL of a water/acetic acid mixture (99:1) was added to 0.5 mL of Rm-saliva. The sample was then loaded onto an SPE column (Bond Elut LRC-C18, Agilent Technologies, Santa Clara, CA, USA) conditioned in advance with 2 mL of ethyl acetate, methanol, and the water/acetic acid mixture (99:1). The column was washed with 2 mL of water and hexane. Next, the Bond Elut DEA (Agilent Technologies) was conditioned with 2 mL of ethyl acetate, and the Bond Elut LRC-C18 described above was connected in series. PGE_2_ was eluted from Bond Elut LRC-C18 with 4 mL of ethyl acetate, and the Bond Elut LRC-C18 was removed. Then, the Bond Elut DEA was washed with 2 mL of the water/acetic acid mixture (99:1). Next, absolute NEXUS (Agilent Technologies) was conditioned with 2 mL of ethyl acetate, methanol, and the water/acetic acid mixture (99:1), and the Bond Elut DEA holding PGE_2_ was connected in series. Elution from Bond Elut DEA was performed with 4 mL of the water/acetic acid mixture (99:1)/methanol (3:2), and this eluate was directly retained in absolute NEXUS. After removing Bond Elut DEA, the absolute NEXUS was centrifuged at about 1500×*g* (3000 rpm) for 10 min for dehydration and eluted with 4 mL of ethyl acetate. The ethyl acetate was evaporated under a gentle stream of nitrogen gas, and the residue was re-dissolved in 200 µL of an ammonium (pH 4.5)/methanol mixture (3:2).

Rm-saliva purified by SPE was further purified by two-step fractionation by HPLC. HPLC (LC 20 series, Shimadzu, Kyoto, Japan) coupled with the fraction collector (FRC-10A, Shimadzu) was used for fractionation. The first step was conducted by size exclusion chromatography using the BioSEC-3 column (3 µm, 100 Å, 4.6 mm × 300 mm, Agilent Technologies). The mobile phases A and B were distilled water and 0.1% formic acid containing acetonitrile (5/95, isocratic mode), and the flow rate was 0.5 mL/min. The SPE purified sample (40 µL) was injected into the HPLC, fractionated every minute, and divided into a total of 10 fractions. The 8–9 min fraction, in which PGE_2_ was eluted, proceeded to the next step. The second step was conducted by reverse-phase chromatography using Cadentza CD-C18 (2.7 µm, 3 × 150 mm; Imtact, Kyoto, Japan). Aliquots of 20 µL of the sample were injected and separated using a linear gradient of distilled water and acetonitrile at a flow rate of 0.3 mL/min. The gradient was programmed as follows: t = 0–2 min, 10% acetonitrile; t = 28 min, 60% acetonitrile; t = 32 min, 95% acetonitrile; and t = 32–35 min, 95% acetonitrile. A fraction was taken every 1 min, and PGE_2_ was eluted in the 23–24 min fraction. The final fraction was dried under a gentle stream of nitrogen gas and re-dissolved in 10 µL of acetonitrile.

### Quantification of PGE_2_ by UPLC-MS/MS

The PGE_2_ concentration in Rm-saliva was quantified by the Agilent 6495B UPLC-MS/MS system (Agilent Technologies). The separation of PGE_2_ was performed on a Poreshell EC-C18 column (2.7 µm, 3 mm, 100 mm; Agilent Technologies) at a flow rate of 1.0 mL/min at 60 °C. Mobile phase A was distilled water, and mobile phase B was methanol containing 0.1% formic acid. The following gradient program was applied: t = 0–1 min, 5% B; t = 10 min, 95% B; t = 11 min, 95% B. The injection volume was 1 µL. Detection was performed in electrospray ionization-positive mode. The triple quadrupole was operated in multiple-reaction monitoring mode by monitoring a quantifier (*m/z* 351 > 271) and qualifier (*m/z* 351 > 315) transition for PGE_2_. Quantitation was achieved by external calibration using a standard curve built from calibration points at 2.5, 5, 10, 25, 50, and 100 ng/mL.

### Immunohistochemical staining

Immunohistochemical assays were performed as previously described with slight modifications^21,33^. Briefly, skin biopsies of a tick bite site (partially engorged *R*. *microplus* female) and a sample of normal skin as a control were performed using a 6-mm biopsy punch, fixed in 10% buffered formalin, and processed routinely by embedding in paraffin wax. The skin sections were then immunohistochemically stained for PGE_2_ and PD-L1 using anti-PGE_2_ polyclonal Ab (ab2318, Abcam, Cambridge, England, UK) and anti-PD-L1 mAb (6C11-3A11, Rat IgG_2a_)^62^. Histological slides were scanned with Nano Zoomer 2.0-RS (Hamamatsu Photonics, Hamamatsu, Japan) and processed in QuPath ver 0.2.1. Scanned slides were opened as Brightfield (H-DAB) in QuPath, DAB and hematoxylin stains were adjusted using the Estimate Stain Vectors function. Immune cells were annotated based on their morphologies and locations to allow QuPath to automatically classify each cell type using the Create Detection Classifiers function.

### Statistics

Statistical significance was analyzed using the Wilcoxon signed-rank test. A *p* value of < 0.05 was considered statistically significant.

## References

[1] Jonsson NN (2006). The productivity effects of cattle tick (*Boophilus microplus*) infestation on cattle, with particular reference to *Bos indicus* cattle and their crosses. Vet. Parasitol..

[2] Cafrune MM, Aguirre DH, Mangold AJ, Guglielmone AA (1995). Experimental studies of the rate of infection of *Boophilus microplus* eggs with *Babesia bovis*. Res. Vet. Sci..

[3] Kocan KM, de la Fuente J, Blouin EF, Coetzee JF, Ewing SA (2010). The natural history of *Anaplasma marginale*. Vet. Parasitol..

[4] Henrioud AN (2011). Towards sustainable parasite control practices in livestock production with emphasis in Latin America. Vet. Parasitol..

[5] Guerrero FD, Lovis L, Martins JR (2012). Acaricide resistance mechanisms in *Rhipicephalus* (*Boophilus*) *microplus*. Rev. Bras. Parasitol. Vet..

[6] Parizi LF, Pohl PC, Masuda A, da Silva Vaz I (2009). New approaches toward anti-*Rhipicephalus* (*Boophilus*) *microplus* tick vaccine. Rev. Bras. Parasitol. Vet..

[7] Šimo L, Kazimirova M, Richardson J, Bonnet SI (2017). The essential role of tick salivary glands and saliva in tick feeding and pathogen transmission. Front. Cell. Infect. Microbiol..

[8] Sajiki Y (2020). Immunosuppressive effects of sialostatin L1 and L2 isolated from the taiga tick *Ixodes persulcatus* Schulze. Ticks Tick Borne Dis..

[9] Coutinho ML (2020). *Rhipicephalus microplus* serpins interfere with host immune responses by specifically modulating mast cells and lymphocytes. Ticks Tick Borne Dis..

[10] Guo X (2009). Inhibition of neutrophil function by two tick salivary proteins. Infect. Immun..

[11] Fredslund F (2008). Structure of and influence of a tick complement inhibitor on human complement component 5. Nat. Immunol..

[12] Lieskovská J (2015). Tick sialostatins L and L2 differentially influence dendritic cell responses to *Borrelia spirochetes*. Parasit. Vectors.

[13] Chmelař J, Kotál J, Langhansová H, Kotsyfakis M (2017). Protease inhibitors in tick saliva: The role of serpins and cystatins in tick-host-pathogen interaction. Front. Cell. Infect. Microbiol..

[14] Kotsyfakis M (2008). Cutting edge: Immunity against a "silent" salivary antigen of the Lyme vector *Ixodes scapularis* impairs its ability to feed. J. Immunol..

[15] Brake DK, Pérez de León AA (2012). Immunoregulation of bovine macrophages by factors in the salivary glands of *Rhipicephalus microplus*. Parasit. Vectors.

[16] Kotál J (2015). Modulation of host immunity by tick saliva. J. Proteomics.

[17] Tirloni L (2016). The putative role of *Rhipicephalus microplus* salivary serpins in the tick-host relationship. Insect Biochem. Mol. Biol..

[18] Xu T, Lew-Tabor A, Rodriguez-Valle M (2016). Effective inhibition of thrombin by *Rhipicephalus microplus* serpin-15 (RmS-15) obtained in the yeast *Pichia pastoris*. Ticks Tick Borne Dis..

[19] Phipps RP, Stein SH, Roper RL (1991). A new view of prostaglandin E regulation of the immune response. Immunol. Today.

[20] Kalinski P (2012). Regulation of immune responses by prostaglandin E_2_. J. Immunol..

[21] Sajiki Y (2018). Prostaglandin E_2_ induction suppresses the Th1 immune responses in cattle with Johne's disease. Infect. Immun..

[22] Ribeiro JM, Makoul GT, Levine J, Robinson DR, Spielman A (1985). Antihemostatic, antiinflammatory, and immunosuppressive properties of the saliva of a tick, *Ixodes dammini*. J. Exp. Med..

[23] Ribeiro JM, Evans PM, MacSwain JL, Sauer J (1992). *Amblyomma americanum*: Characterization of salivary prostaglandins E_2_ and F_2α_ by RP-HPLC/bioassay and gas chromatography-mass spectrometry. Exp. Parasitol..

[24] Bowman AS, Sauer JR, Zhu K, Dillwith JW (1995). Biosynthesis of salivary prostaglandins in the lone star tick, *Amblyomma americanum*. Insect Biochem. Mol. Biol..

[25] Oliveira CJ (2011). Deconstructing tick saliva: Non-protein molecules with potent immunomodulatory properties. J. Biol. Chem..

[26] Sá-Nunes A (2007). Prostaglandin E_2_ is a major inhibitor of dendritic cell maturation and function in *Ixodes scapularis* saliva. J. Immunol..

[27] Poole NM, Mamidanna G, Smith RA, Coons LB, Cole JA (2013). Prostaglandin E_2_ in tick saliva regulates macrophage cell migration and cytokine profile. Parasit. Vectors.

[28] Esteves E (2019). *Amblyomma sculptum* salivary PGE_2_ modulates the dendritic cell-*Rickettsia rickettsia* interactions in vitro and in vivo. Front. Immunol..

[29] Dickinson RG, O'Hagan JE, Schotz M, Binnington KC, Hegarty MP (1976). Prostaglandin in the saliva of the cattle tick *Boophilus microplus*. Aust. J. Exp. Biol. Med. Sci..

[30] Inokuma H, Kemp DH, Willadsen P (1994). Prostaglandin E_2_ production by the cattle tick (*Boophilus microplus*) into feeding sites and its effect on the response of bovine mononuclear cells to mitogen. Vet. Parasitol..

[31] Prima V, Kaliberova LN, Kaliberov S, Curiel DT, Kusmartsev S (2017). COX2/mPGES1/PGE_2_ pathway regulates PD-L1 expression in tumor-associated macrophages and myeloid-derived suppressor cells. Proc. Natl. Acad. Sci. U.S.A..

[32] Wang J, Zhang L, Kang D, Yang D, Tang Y (2018). Activation of PGE_2_/EP2 and PGE_2_/EP4 signaling pathways positively regulate the level of PD-1 in infiltrating CD8^+^ T cells in patients with lung cancer. Oncol. Lett..

[33] Goto S (2020). Upregulation of PD-L1 expression by prostaglandin E_2_ and the enhancement of IFN-γ by anti-PD-L1 antibody combined with a COX-2 inhibitor in *Mycoplasma bovis* infection. Front. Vet. Sci..

[34] Okazaki T, Honjo T (2007). PD-1 and PD-1 ligands: From discovery to clinical application. Int. Immunol..

[35] Mejri N, Rutti B, Brossard M (2002). Immunosuppressive effects of *Ixodes ricinus* tick saliva or salivary gland extracts on innate and acquired immune response of BALB/c mice. Parasitol. Res..

[36] Brossard M, Wikel SK (2004). Tick immunobiology. Parasitology.

[37] Konnai S, Murata S, Ohashi K (2017). Immune exhaustion during chronic infections in cattle. J. Vet. Med. Sci..

[38] Tirloni L (2019). *Amblyomma americanum* serpin 27 (AAS27) is a tick salivary anti-inflammatory protein secreted into the host during feeding. PLoS Negl. Trop. Dis..

[39] Salát J (2010). Crystal structure and functional characterization of an immunomodulatory salivary cystatin from the soft tick *Ornithodoros moubata*. Biochem. J..

[40] Schwarz A, Valdés JJ, Kotsyfakis M (2012). The role of cystatins in tick physiology and blood feeding. Ticks Tick Borne Dis..

[41] Aounallah H (2020). Tick salivary compounds for targeted immunomodulatory therapy. Front. Immunol..

[42] Martins LA, Kotál J, Bensaoud C, Chmelař J, Kotsyfakis M (2020). Small protease inhibitors in tick saliva and salivary glands and their role in tick-host-pathogen interactions. Biochim. Biophys. Acta Proteins Proteom..

[43] Sajiki Y (2019). Prostaglandin E_2_-induced immune exhaustion and enhancement of antiviral effects by anti-PD-L1 antibody combined with COX-2 inhibitor in Bovine leukemia virus infection. J. Immunol..

[44] Moré DD (2019). Network analysis uncovers putative genes affecting resistance to tick infestation in Braford cattle skin. BMC Genom..

[45] Preston SG (2013). Novel immunomodulators from hard ticks selectively reprogramme human dendritic cell responses. PLoS Pathog..

[46] Carvalho-Costa TM (2015). Immunosuppressive effects of *Amblyomma cajennense* tick saliva on murine bone marrow-derived dendritic cells. Parasit. Vectors.

[47] Yun SJ (2019). Regulation of TIM-3 expression in a human T cell line by tumor-conditioned media and cyclic AMP-dependent signaling. Mol. Immunol..

[48] Goto S (2017). Increase of cells expressing PD-1 and PD-L1 and enhancement of IFN-γ production via PD-1/PD-L1 blockade in bovine mycoplasmosis. Immun. Inflamm. Dis..

[49] Konnai S (2013). Enhanced expression of LAG-3 on lymphocyte subpopulations from persistently lymphocytotic cattle infected with bovine leukemia virus. Comp. Immunol. Microbiol. Infect. Dis..

[50] Kazimírová M, Štibrániová I (2013). Tick salivary compounds: Their role in modulation of host defences and pathogen transmission. Front. Cell. Infect. Microbiol..

[51] Wikel SK (1999). Tick modulation of host immunity: An important factor in pathogen transmission. Int. J. Parasitol..

[52] Schoeler GB, Wikel SK (2001). Modulation of host immunity by haematophagous arthropods. Ann. Trop. Med. Parasitol..

[53] Zeidner N, Dreitz M, Belasco D, Fish D (1996). Suppression of acute *Ixodes scapularis* induced *Borrelia burgdorferi* infection using tumor necrosis factor-alpha, interleukin-2, and interferon-gamma. J. Infect. Dis..

[54] Han S (2008). Rapid deletion of antigen-specific CD4^+^ T cells following infection represents a strategy of immune evasion and persistence for *Anaplasma marginale*. J. Immunol..

[55] Okagawa T (2016). Cooperation of PD-1 and LAG-3 contributes to T-cell exhaustion in *Anaplasma marginale*-infected cattle. Infect. Immun..

[56] Wei N (2020). Inclusion of PD-L1 into a recombinant profilin antigen enhances immunity against *Babesia microti* in a murine model. Ticks Tick Borne Dis..

[57] Reck J (2009). Systemic alterations of bovine hemostasis due to *Rhipicephalus* (*Boophilus*) *microplus* infestation. Res. Vet. Sci..

[58] Clarke RH, Hewetson RW (1971). A modification to the collection of saliva from *Boophilus microplus*. J. Parasitol..

[59] Ciprandi A, de Oliveira SK, Masuda A, Horn F, Termignoni C (2006). *Boophilus microplus*: Its saliva contains microphilin, a small thrombin inhibitor. Exp. Parasitol..

[60] Nishimori A (2017). In vitro and in vivo antivirus activity of an anti-programmed death-ligand 1 (PD-L1) rat-bovine chimeric antibody against bovine leukemia virus infection. PLoS ONE.

[61] Ikebuchi R (2013). Blockade of bovine PD-1 increases T cell function and inhibits bovine leukemia virus expression in B cells in vitro. Vet. Res..

[62] Ikebuchi R (2014). Influence of PD-L1 cross-linking on cell death in PD-L1-expressing cell lines and bovine lymphocytes. Immunology.

[63] Komaba J (2009). Development and validation of an on-line two-dimensional reversed-phase liquid chromatography-tandem mass spectrometry method for the simultaneous determination of prostaglandins E_2_ and F_2α_ and 13,14-dihydro-15-keto prostaglandin F_2α_ levels in human plasma. Biomed. Chromatogr..

